# Sepsis 2016 Agra, India: Agra, India. 5-6 February 2016

**DOI:** 10.1186/s13054-016-1204-x

**Published:** 2016-03-02

**Authors:** Surinder Kumar Sharma, Anurag Rohatgi, Mansi Bajaj, Charles L. Sprung, Ricardo Calderon Morales, Harvey Kasdan, Allon Reiter, Tobias Volker, Julien Meissonnier, Natalia Beloborodova, Viktor Moroz, Aleksandra Bedova, Yulia Sarshor, Artem Osipov, Katerina Chernevskaya, Nadezhda Fedotcheva, Ekaterina Chernevskaya, Natalia Beloborodova, Hisashi Imahase, Kosuke C. Yamada, Yuichiro Sakamoto, Miho Ohta, Ryota Sakurai, Mayuko Yahata, Mitsuru Umeka, Toru Miike, Hiroyuki Koami, Futoshi Nagashima, Takashi Iwamura, Satoshi Inoue, Zhifeng Li, Dennis Grech, Patrick Morcillo, Alex Bekker, Luis Ulloa, Samanwoy Mukhopadhyay, Abhay D. Pandey, Samsiddhi Bhattacharjee, Saroj K. Mohapatra, Julie K. Wilson, Savita Jadhav, Rabindra Nath Misra, Nageswari Gandham, Kalpana Angadi, Chanda Vywahare, Neetu Gupta, Deepali Desai, Anahita Bakochi, Tirthankar Mohanty, Adam Linder, Johan Malmström, Dimple Anand, Seema Bhargava, Lalit Mohan Srivastava, Sumit Ray, Jane Fisher, Peter Bentzer, Adam Linder, Luis Henrique Angenendt da Costa, Nilton Nascimentos dos Santos Júnior, Carlos Henrique Rocha Catalão, Maria José Alves da Rocha, Alfredo Focà, Cinzia Peronace, Giovanni Matera, Aida Giancotti, Giorgio Settimo Barreca, Angela Quirino, Maria Teresa Loria, Pio Settembre, Maria Carla Liberto, Bruno Amantea, Christiane Hartog, Claudia Moeller, Carolin Fleischmann, Daniel Thomas-Rueddel, Vlasislav Vlasakov, Bram Rochwerg, Philip Theurer, Konrad Reinhart, Anna E. Smith, Sandra D. Taylor, Christopher Da Costa, Amanda Radford, Terry Lee, Joel Singer, John Boyd, David Fineberg, Mark Williams, James A. Russell

**Affiliations:** Department of General Medicine, Lady Hardinge Medical College, Delhi, India; Department of Anesthesiology and Critical Care Medicine, Hadassah Hebrew University Medical Center, Jerusalem, Israel; LeukoDx, Jerusalem, Israel; Negovsky Research Institute of General Reanimatology, Moscow, Russia; Institute of Theoretical and Experimental Biophysics, Russian Academy of Sciences, Pushchino, Moscow Region Russia; Negovsky Research Institute of General Reanimatology, Moscow, Russia; Emergency and Critical Care Center, Saga University Hospital, Saga City, Japan; Department of Surgery and Anesthesiology, New Jersey Medical School, Rutgers University, Newark, NJ 07103 USA; National Institute of Biomedical Genomics, Kalyani, 741251 India; Critical Care and Anaesthetics, Royal Marsden Hospital, London, UK; Department of Microbiology, Dr. D.Y.Patil Medical College, Hospital and research Centre ( Dr D.Y.Patil Vidyapeeth Pune), Pimpri-Pune, 411018 India; Department of Clinical Sciences, Division of Infection Medicine, BMC B14, Lund University, SE-221 85 Lund, Sweden; Department of Biochemistry, Sir Ganga Ram Hospital, New Delhi, 110060 India; Department of Critical Care and Emergency Medicine, Sir Ganga Ram Hospital, New Delhi, 110060 India; Department of Clinical Sciences, Division of Infection Medicine, BMC B14, Lund University, SE-221 85 Lund, Sweden; Department of Anesthesia and Intensive Care, Skåne University Hospital, SE-221 85 Lund, Sweden; Department of Neurosciences and Behavioral Sciences, Ribeirão Preto Medical School, University of São Paulo, Ribeirão Preto, Brazil; Department of Morphology, Physiology and Basic Pathology, School of Dentistry of Ribeirão Preto, University of São Paulo, Ribeirão Preto, Brazil; Institute of Microbiology, Department of Health Sciences, University “Magna Graecia” of Catanzaro, Catanzaro, Italy; Intensive Care Unit, Department of Health Sciences, “Magna Graecia” University of Catanzaro, Catanzaro, Italy; Department for Anesthesiology and Intensive Care, Jena University Hospital, Jena, Germany; Center for Sepsis Control and Care, Jena University Hospital, Jena, Germany; Department of Medicine (Division of Critical Care), McMaster University, Hamilton, Ontario Canada; Department of Veterinary Clinical Sciences, College of Veterinary Medicine, Purdue University, West Lafayette, Indiana USA; Asahi Kasei Pharma America, Waltham, MA USA; Centre for Health Evaluation and Outcome Science (CHEOS), St. Paul’s Hospital, University of British Columbia, 1081 Burrard Street, Vancouver, BC V6Z 1Y6 Canada; Centre for Heart Lung Innovation, St. Paul’s Hospital, University of British Columbia, 1081 Burrard Street, Vancouver, BC V6Z 1Y6 Canada; Division of Critical Care Medicine St. Paul’s Hospital, University of British Columbia, 1081 Burrard Street, Vancouver, BC V6Z 1Y6 Canada

## Abstract

P1 D-Dimer in adult patients with presumed sepsis and their clinical outcomes

Surinder Kumar Sharma, Anurag Rohatgi, Mansi Bajaj

P2 Diagnosis of infection utilizing Acellix CD64

Charles L. Sprung, Ricardo Calderon Morales, Harvey Kasdan, Allon Reiter, Tobias Volker, Julien Meissonnier

P3 High levels of phenylcarboxylic acids reflect the severity in ICU patients and affect phagocytic activity of neutrophils

Natalia Beloborodova, Viktor Moroz, Aleksandra Bedova, Yulia Sarshor, Artem Osipov, Katerina Chernevskaya

P4 The role of bacterial phenolic metabolites in mitochondrial dysfunction

Nadezhda Fedotcheva, Ekaterina Chernevskaya, Natalia Beloborodova

P5 The early diagnosis of severe sepsis and judgment of rapid transport to critical care center: better prognostic factor

Hisashi Imahase, Kosuke C Yamada, Yuichiro Sakamoto, Miho Ohta, Ryota Sakurai, Mayuko Yahata, Mitsuru Umeka, Toru Miike, Hiroyuki Koami, Futoshi Nagashima, Takashi Iwamura, Satoshi Inoue

P6 Translational neuromodulation of the immune system

Zhifeng Li, Dennis Grech, Patrick Morcillo, Alex Bekker, Luis Ulloa

P7 Pathway-level meta-analysis reveals transcriptional signature of septic shock

Samanwoy Mukhopadhyay, Abhay D Pandey, Samsiddhi Bhattacharjee, Saroj K Mohapatra

P8 Antibiotic dosing in septic patients on the critical care unit - a literature review

Julie K Wilson

P9 Pandemic of *Escherichia coli* clone O25: H4-ST131 producing CTX-M-15 extended spectrum- β- lactamase- as serious cause of multidrug resistance extraintestinal pathogenic *E. coli* infections in India

Savita Jadhav, Rabindra Nath Misra, Nageswari Gandham, Kalpana Angadi, Chanda Vywahare, Neetu Gupta, Deepali Desai

P10 Detection and characterization of meningitis using a DDA-based mass spectrometry approach

Anahita Bakochi, Tirthankar Mohanty, Adam Linder, Johan Malmström

P11 Diagnostic usefulness of lipid profile and procalcitonin in sepsis and trauma patients

Dimple Anand, Seema Bhargava, Lalit Mohan Srivastava, Sumit Ray

P12 Heparin – a novel therapeutic in sepsis?

Jane Fisher, Peter Bentzer, Adam Linder

P13 Hypothalamic impairment is associated with vasopressin deficiency during sepsis

Luis Henrique Angenendt da Costa, Nilton Nascimentos dos Santos Júnior Carlos Henrique Rocha Catalão, Maria José Alves da Rocha

P14 Presepsin (soluble CD14 subtype) is a dependable prognostic marker in critical septic patients

Alfredo Focà, Cinzia Peronace, Giovanni Matera, Aida Giancotti, Giorgio Settimo Barreca, Angela Quirino, Maria Teresa Loria, Pio Settembre, Maria Carla Liberto, Bruno Amantea

P15 Safety and efficacy of gelatin-containing solutions versus crystalloids and albumin - a systematic review with quantitative and qualitative summaries

Christiane Hartog, Christiane Hartog, Claudia Moeller, Carolin Fleischmann, Daniel Thomas-Rueddel, Vlasislav Vlasakov, Bram Rochwerg, Philip Theurer, Konrad Reinhart

P16 Immunomodulatory properties of peripheral blood mesenchymal stem cells following endotoxin stimulation in an equine model

Anna E. Smith, Sandra D. Taylor

P17 Frequency and outcome of early sepsis-associated coagulopathy

Christopher Da Costa, Amanda Radford, Terry Lee, Joel Singer, John Boyd, David Fineberg, Mark Williams, James A Russell

## P1 D-Dimer in adult patients with presumed sepsis and their clinical outcomes

### Surinder Kumar Sharma, Anurag Rohatgi, Mansi Bajaj

#### Department of General Medicine, Lady Hardinge Medical College, Delhi, India

##### **Correspondence:** Mansi Bajaj (bajaj.manc@gmail.com) – Department of General Medicine, Lady Hardinge Medical College, Delhi, India

**Background:** The tools are currently limited in predicting which patients with an infection will progress to severe sepsis, shock, or death. The Systemic Inflammatory Response Syndrome (SIRS) criteria, while part of the definition of sepsis, are not adequately sensitive or specific to be used alone to predict the clinical course of a patient [1]. A predictive biomarker could be helpful to clinicians to risk-stratify infected patients to an appropriate level of care. As a candidate biomarker of sepsis, fibrin D-dimer has demonstrated sensitivity for sepsis in ICU patients, however limiting application of the data to Emergency patients [2,3]. If the correlation of D-dimer levels with illness severity described in ICU patients could be reproduced in the Emergency population, the D-dimer could be used to better risk stratify patients with infections into appropriate levels of care [4]. The aim was to study the level of D-dimer in patients with presumed sepsis and the prevalence of organ dysfunction, death and intensive care unit (ICU) admission in patients with presumed sepsis with D-dimer levels > = 0.5 μg/ml(FEU).

**Materials and methods:** Sixty adult patients (18 years and above) presenting to the Emergency, from November 2012 and march 2014, with a suspected infection (radiographic, laboratory, or clinical findings indicating a need for antibiotics) and at least two out of four SIRS criteria excluding patients with a history of thromboembolic disease, recent surgery, renal disease, malignancy, pregnant women were studied prospectively in an observational study and evaluated by a semi-quantitative D-dimer assay and Sepsis-related Organ Failure Assessment (SOFA) score on day 0, 2 and 30.Observations were made regarding Admission to In-Patient Ward, Average length of stay (days), ICU Admission, Average ICU stay (days), Organ Dysfunction in the Emergency, Organ Dysfunction at 48 hours (only for In-Patients), In-hospital death, Organ Dysfunction during 30 day follow up (only for In-Patients), 30-Day Mortality Rate, 30-Day Survival Rate. Association between D-Dimer levels and organ dysfunction, ICU admission and mortality was evaluated on day 0 and day 2. Correlation between D-dimer levels and Sofa score was evaluated on day 0 and day 2.

**Fig. 1:**
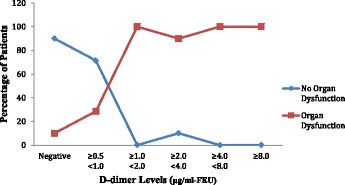
**(abstract P1).** Distribution of Organ Dysfunction across the range of D-dimer levels on day 0. (n = 60)

**Fig. 2:**
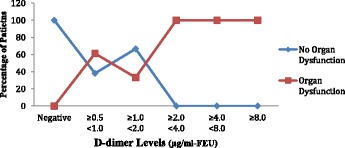
**(abstract P1).** Distribution of Organ Dysfunction across the range of D-dimer levels on day 2. (n = 60)

**Results**: All the 60 patients (i.e. 100 %) were admitted in In-Patient Wards. The mean length of in-patient stay of patients was 7.43 days (±4.07) ranging from a minimum of 1 day to 32 days. Among the 60 patients analysed, 21 patients (35 %) required ICU Admission. The mean length of ICU stay of patients was 4.87 days (±6.08) ranging from a minimum of 1 day to 29 days. Out of 60, 36 patients (60 %) presented with organ dysfunction in Emergency, 19 patients (31.7 %) had organ dysfunction at 48 hours and 1 patient (1.7 %) had succumbed to death by 48 hours. The In-hospital Mortality and 30 Day Mortality was the same 4 out of 60 patients (6.7 %). The 30-day survival rate was found to be 93.3 %. The higher the D-dimer levels on day 0 and day 2, the higher the percentage of patients with organ dysfunction on that respective day (Figs. 1 and 2).

**Fig. 3:**
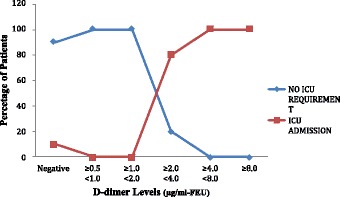
**(abstract P1).** Distribution of ICU requirement across the range of D-dimer levels on day 0. (n = 60)

**Fig. 4:**
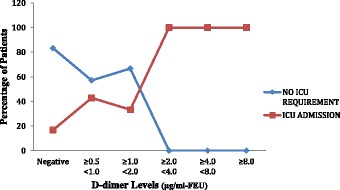
**(abstract P1).** Distribution of ICU admission across the range of D-dimer levels on day 2. (n = 60)

The higher the D-dimer levels on day 0 and day 2, the higher the percentage of patients who require ICU admission during 30 day period (Figs. 3 and 4).

**Fig. 5:**
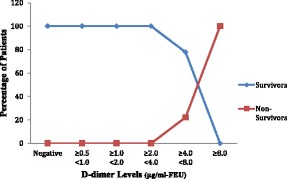
**(abstract P1).** Distribution of Mortality across the range of D-dimer levels on day 0. (n = 60)

**Fig. 6:**
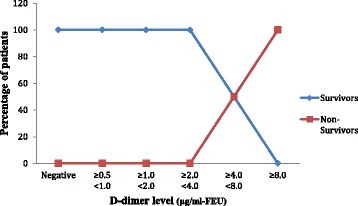
**(abstract P1).** Distribution of Mortality across the range of D-dimer levels on day 2. (n = 60)

The higher the D-dimer on day 0 and day 2, the higher the percentage of patients who succumbed to death during 30-day period (Figs. 5 and 6).

**Fig. 7:**
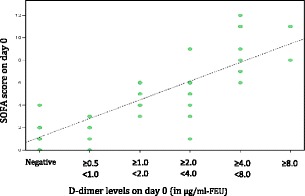
**(abstract P1).** Scatter Plot showing SOFA Score on Day 0 as a function of D-dimer levels on Day 0

**Fig. 8:**
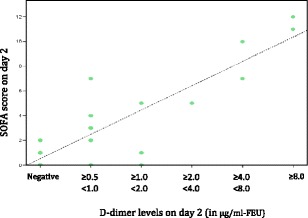
**(abstract P1).** Scatter Plot showing SOFA Scores at 48 hrs (Day 2) as a function of D-dimer levels at 48 hrs (Day 2)

In patients with presumed sepsis, 66.7 % patients had positive D-dimer levels on day 0 which was found to have a statistically significant association with organ dysfunction at presentation (p value <0.001) and ICU admission (p value <0.01), respectively. However, the association of D-dimer levels on day 0 with mortality was not statistically significant (p value ≥ 0.05). On day 2, positive D-dimer levels were demonstrated to have a statistically significant association with organ dysfunction on day 2 (p value <0.001) and ICU admission (p value <0.01), respectively. However, the association of D-dimer levels on day 2 with mortality was not statistically significant (p value ≥ 0.05). A positive strong correlation was demonstrated between D-dimer levels and SOFA Scores both on Day 0 (Pearson’s correlation coefficient r = .850, p < .001) and Day 2 (Pearson’s correlation coefficient r = .870, p < .001) (Figs. 7 and 8).

**Conclusions:** It can be concluded that fibrin D-dimer is a promising biomarker that may identify, in a simple and rapid way, infected patients who are at increased risk for organ dysfunction, ICU admission and death; thus helping in triage. These patients, consequently, could require special attention upon admission to the emergency service.

## P2 Diagnosis of infection utilizing Acellix CD64

### Charles L. Sprung^1^, Ricardo Calderon Morales^1^, Harvey Kasdan^2^, Allon Reiter^2^, Tobias Volker^2^, Julien Meissonnier^2^

#### ^1^Department of Anesthesiology and Critical Care Medicine, Hadassah Hebrew University Medical Center, Jerusalem, Israel; ^2^LeukoDx, Jerusalem, Israel

##### **Correspondence:** Charles L. Sprung (areiter@leukodx.com) – Department of Anesthesiology and Critical Care Medicine, Hadassah Hebrew University Medical Center, Jerusalem, Israel

**Background**: Differentiating patients who are infected or not in the intensive care unit (ICU) can be very difficult. Present diagnostic tests remain inadequate. CD64 has been found to be a potentially useful marker to identify infected patients. Unfortunately, CD64 measured by standard flow cytometers in a laboratory takes hours to perform. The Accellix table top flow cytometer automates the process: sample preparation and reading are performed in a dedicated disposable cartridge, and analytical data processing utilizing proprietary algorithms provides answers directly to the user. The purpose of this study was to evaluate the Accellix CD64 instrument, which provides results in 20 minutes in ICU patients with and without infections.

**Fig. 9:**
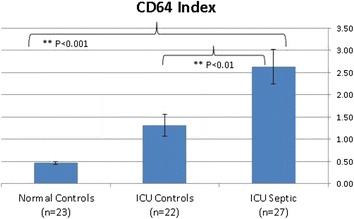
**(abstract P2).**

**Materials and methods:** The Accellix disposable cartridge-based platform implements sample preparations using three reagent blisters. The three Accellix CD64 cartridge blisters contain staining cocktail of conjugated monoclonal antibodies, lysis buffer and reference beads respectively. Once sample processing is complete, the sample flows through a dedicated reading channel where data is acquired. Infected (ICUi) and non-infected ICU patients (ICU Control-ICUc) and normal volunteers (C) had CD64 levels measured by the Accellix CD64 instrument. Measurements were calculated as ‘CD64 index’, i.e. the ratio between the fluorescence of the PMN population and the fluorescence of control beads. ICU infection, ICU control and normal control patients’ results can be seen in Fig. 9.

**Results**: 72 subjects were studied (ICUi- 27, ICUc-22 and C-23). CD64 Index levels were higher (mean ± SEM) in ICU infection patients then ICU control and normal control patients (2.62 ± 0.39 vs. 1.31 ± 0.24 vs. 0.46 ± 0.04, p < 0.01 for ICUi vs. ICUc, p < 0.001 for ICUi vs. C).

**Conclusions:** CD64 Index levels are higher in infected than non-infected ICU patients. Accellix CD64 is a promising instrument to differentiate infected from non-infected ICU patients in a timely manner.

## P3 High levels of phenylcarboxylic acids reflect the severity in ICU patients and affect phagocytic activity of neutrophils

### Natalia Beloborodova, Viktor Moroz, Aleksandra Bedova, Yulia Sarshor, Artem Osipov, Katerina Chernevskaya

#### Negovsky Research Institute of General Reanimatology, Moscow, Russia

##### **Correspondence:** Natalia Beloborodova (nvbeloborodova@yandex.ru) – Negovsky Research Institute of General Reanimatology, Moscow, Russia

**Background**: Previously it has been shown that phenylcarboxylic acids (PhCAs) could have microbial origin in human blood [1], associated with mortality [2] and demonstrated biological activity [3]. It is known that neutrophil dysfunction is one of the key mechanisms of severe infection and sepsis development.

**Objectives**: to determine PhCAs levels typical for different severity of bacterial infection and the ability of these clinically relevant concentrations of PhCAs to affect the neutrophil phagocytosis *in-vitro*.

**Materials and methods**: Blood samples were collected from outpatients (n = 17) with local bacterial infection of skin and soft tissue (ISST) and from critically ill patients with documented infection (n = 60): 46 patients on the day of admission to ICU and 14 - in terminal state one day before death. Clinical and laboratory data, SOFA Scores in patients were matched. Serum levels of PhCAs were measured using gas chromatography with flame ionization detector. Data from the previous study in healthy adult donors (n = 72) were used as a control [4]. The effect of PhCAs on the function of peripheral blood neutrophils was assessed by measuring the number of latex beads (d = 1.5 μm) phagocytized *in-vitro*, blood samples from healthy volunteers (n = 30). Data were compared by Mann–Whitney U-test and Sign test, Spearman's rank correlation coefficient was defined, p < 0.05 was considered significant (STATISTICA 10).

**Fig. 10:**
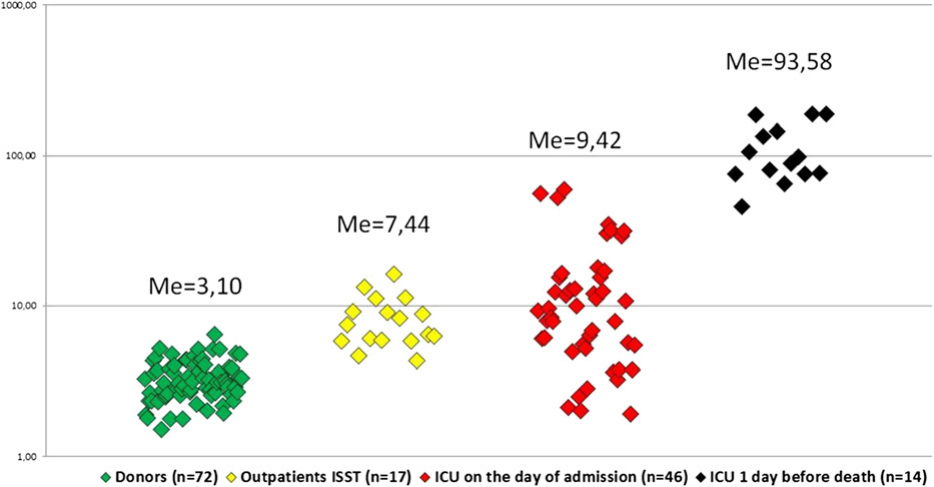
**(abstract P3).** Total level of PhCAs, μM

**Results**: Levels of PhLA, p-HPhLA and p-HPhAA in patients were significantly higher than in healthy donors, as in severe infections and local bacterial inflammatory processes (Fig. 10, Table 1).

A positive correlation with serum levels of the PhCAs and SOFA were found (r = 0.645-0.740, p < 0.001). In vitro we observed a significant decrease in the number of phagocytic neutrophils by 15 % under the influence of 6.0 μM p-HPhAA and 6.0 μM PhLA. Intensity of the absorption capacity of neutrophils significantly decreased by: 14 %, 31 % under the influence of 0.6, 6.0 μM p-HPhLA, respectively; 23 % - 0.6 μM PhLA; 31 %, 30 % - 0.6, 6.0 μM p-HPhAA, respectively. Perhaps, the ability of bacteria to produce PhCAs is one of adaptive mechanisms to protect from effector cells of immune system.

**Conclusions**: Serum levels of PhCAs reflect the severity in ICU’s patients and reach maximum values in terminally ill patients with infection. *In-vitro* PhCAs are able to inhibit phagocytic activity of neutrophils in clinically significant concentrations.

**Acknowledgements**

Supported by Russian Science Foundation Grant №15-15-00110.

**References**

1. Beloborodova NV, Khodakova AS, Bairamov IT, Olenin AYu: Microbial origin of phenylcarboxylic acids in the human body. Biochemistry (Mosc). 2009,74(12):1350–1355.

2. Rogers AJ, McGeachie M, Baron RM, Gazourian L. et al. Metabolomic derangements are associated with mortality in critically ill adult patients. PLoS One. 2014 Jan 30; 9(1):e87538

3. Fedotcheva, N.I., Kazakova R.E., Kondrashova M.N., Beloborodova N.V. Toxic effects of microbial phenolic acids on the functions of mitochondria. Toxicology Letters. 2008.180(3):182–188

4. Beloborodova NV , Moroz V V , Osipov A A , Bedova A Yu et al. Normal level of sepsis-associated phenylcarboxylic acids in human serum. Biochemistry (Moscow) 2015; 80(3):374–378

**Table 1:** **(abstract P3).** Baseline clinical, laboratory characteristics and levels of PhCAs, Median (25-75 %)

Сharacteristics	1. Outpatients ISST	2. on the day of admission to ICU	3. one day before death in ICU	*p*	
				1 vs 2	2 vs 3
N (M/F)	17 (10/7)	46 (26/20)	14 (10/4)	-	-
Age, years	48 (38–63)	58 (48–71)	62 (53–67)	0.103	0.521
WBC, х10^9^/L	11.7 (10.2-12.6)	13.2 (9.8-16.0)	10.4 (6.4-17.2)	0.321	0.874
t, °C	37.3 (37.2-37.6)	37.4 (37.0-38.5)	39.7 (38.9-42.0)	0.944	0.003
Heart rate, bpm	80 (74–82)	109 (98–120)	117 (101–130)	<0.001	0.406
SOFA score	-	7 (3–9)	14 (12–16)	-	<0.001
PhLA, μM	0.86 (0.72-1.55)	1.18 (0.83-2.12)	12.57 (6.69-17.57)	0.242	<0.001
p-HPhLA, μM	3.38 (2.53-4.08)	3.61 (1.21-7.75)	35.74 (24.19-54.59)	0.716	<0.001
p-HPhAA, μM	2.99 (2.40-3.51)	3.34 (2.43-4.93)	28.15 (17.32-44.07)	0.969	<0.001

## P4 The role of bacterial phenolic metabolites in mitochondrial dysfunction

### Nadezhda Fedotcheva^1^, Ekaterina Chernevskaya^2^, Natalia Beloborodova^2^

#### ^1^Institute of Theoretical and Experimental Biophysics, Russian Academy of Sciences, Pushchino, Moscow region, Russia; ^2^Negovsky Research Institute of General Reanimatology, Moscow, Russia

**Background**: Mitochondrial dysfunction is inherent in many systemic pathologies, including the inflammatory syndrome and sepsis. It is assumed that mitochondrial dysfunction in sepsis is associated with the overproduction of cytokines, reactive oxygen species (ROS), and NO, which affects several enzymes and complexes of the respiratory chain. However, the role of bacterial metabolites, which can accumulate in the blood of patients with infection, is not considered. Differences in the serum level of phenolic metabolites, in septic patients, compared with healthy donors was shown earlier [1]. It was found that these compounds affect the mitochondrial respiration and ROS production, showing either the prooxidant or antioxidant effects depending on their chemical structure [2,3]. In this work we studied the effect of bacterial phenolic acids on the activity of mitochondrial oxidative enzymes in the liver and kidney.

**Materials and methods**: The experiments were performed on male Wistar rats. Liver mitochondria were isolated by the standard method. Concentrated homogenates (1 g tissue/ml medium) were prepared from the liver and kidney by a rapid procedure involving cooling, punching through press, and homogenization in a medium (125 mM KCl, 30 mM HEPES, pH 7.4). The effect of phenolic acids (all from Sigma) on the oxidation activity of mitochondria (0.5 mg protein/ml) and homogenate (1 mg protein/ml) was assessed by the reduction of nitroblue tetrazolium (NBT) at a wavelength of 560 nm after a 10-min incubation in the presence of an oxidation substrate followed by the addition of 20 μl of 10 % Triton X-100.

**Fig. 11:**
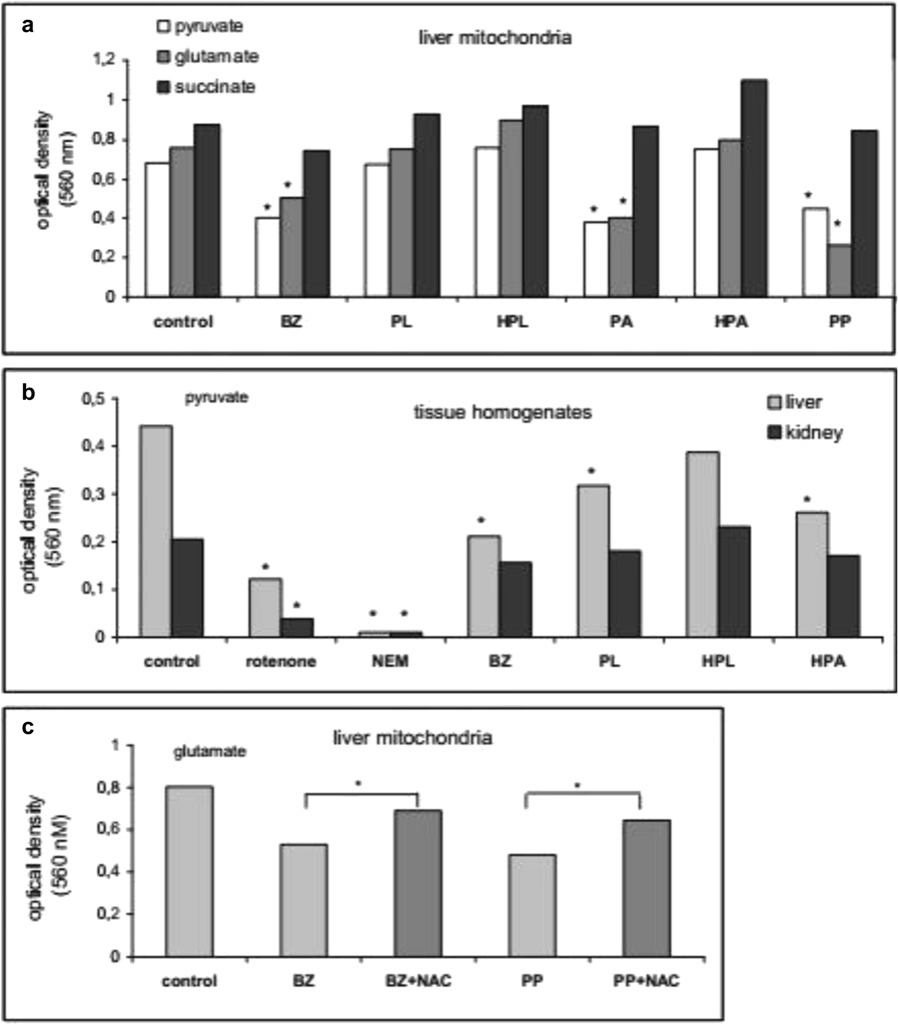
**(abstract P4).** Influence of phenolic acids on oxidative metabolism in liver mitochondria (**a**), tissue homogenates (**b**) and removing their inhibitory effect by N-acetylcysteine (**c**). Optical density of reduced NBT in the presence of 5 mM substrate and 100 μM phenolic acid – benzoic (BZ), phenyllactic (PL), p-hydroxyphenyllactic (HPL), phenylacetic (PA), p-hydroxyphenylacetic and phenylpropionic (PP) acids is presented. Inhibitors of NAD-dependent oxidation rotenone and N-ethylmaleimide (NEM) confirm the substrate specificity of NBT reduction. Antioxidant N-acetylcysteine (1 mM) prevents the inhibition induced by phenolic acids. * р < 0,05 compared with the corresponding control.

**Results**: Phenolic acids influenced mitochondrial oxidative activity differently depending on the chemical structure of phenolic compounds and the oxidation substrate. Benzoic, phenylacetic, and phenylpropionic acids inhibited NAD-dependent oxidation by 40 % and weakly decreased succinate oxidation, while *p*-hydroxyphenyllactate and *p*-hydroxyphenylacetate activated NBT reduction supported by succinate and glutamate oxidation (Fig. 11). Similar effects were detected on tissue homogenates and were more pronounced in the liver than in the kidney. The antioxidant N-acetylcysteine prevented inhibition, indicating the contribution of thiol groups and ROS production to the decrease in oxidative activity induced by phenolic acids.

**Fig. 12:**
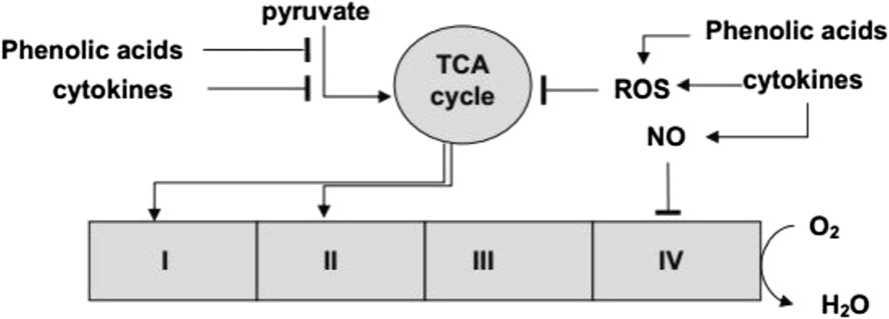
**(abstract P4).** Contribution of phenolic acids and cytokines to the mitochondrial dysfunction. Cytokines indirectly (through signaling molecules) inhibit pyruvate dehydrogenase and induce NO and ROS production. Some of bacterial phenolic acid directly inhibit NAD-dependent oxidation and regulate ROS production

**Conclusions**: Aromatic bacterial metabolites can be involved in the development of the mitochondrial failure. Some phenolic acids inhibit NAD-dependent oxidation. Their action is tissue-specific. The influence of these phenolic acids is similar in some respect to the action of proinflammatory cytokines (Fig. 12). Thus, phenolic acids may regulate the signaling action of interleukins by affecting the respiration and ROS production in mitochondria and neutrophils [3].

**Acknowledgements**

Supported by Russian Science Foundation Grant №15-15-00110.

**References**

1. Khodakova, A., Beloborodova, N. 2007. Microbial metabolites in the blood of patients with sepsis. Crit. Care. 2007, 11(Suppl 4), P5 doi:10.1186/cc5984

2. N. I. Fedotcheva, R. E. Kazakov, M. N. Kondrashova, N. V. Beloborodova. Toxic Effects of Microbial Phenolic Acids on the Functions of Mitochondria. Toxycology Letters, 2008, 180: 182–188 doi:10.1016/j.toxlet.2008.06.861

3. N. Beloborodova, I. Bairamov, A. Olenin, V. Shubina, V. Teplova, N. Fedotcheva. Effect of phenolic acids of microbial origin on production of reactive oxygen species in mitochondria and neutrophils. Journal of Biomedical Science 2012, 19:89 doi:10.1186/1423-0127-19-89

## P5 The early diagnosis of severe sepsis and judgment of rapid transport to critical care center: better prognostic factor

### Hisashi Imahase, Kosuke C Yamada, Yuichiro Sakamoto, Miho Ohta, Ryota Sakurai, Mayuko Yahata, Mitsuru Umeka, Toru Miike, Hiroyuki Koami, Futoshi Nagashima, Takashi Iwamura, Satoshi Inoue

#### Emergency and Critical Care Center, Saga University Hospital, Saga city, Japan

##### **Correspondence:** Hisashi Imahase (hisa.lefty@gmail.com) – Emergency and Critical Care Center, Saga University Hospital, Saga city, Japan

**Background**: The prognosis of patients with severe sepsis or septic shock is improving. In our center, although promoting the standardization and individualization of sepsis treatment, some patients die. We examined the better factor of the prognosis of septic patients.

**Materials and methods**: We included the patients with severe sepsis and septic shock who admitted from the emergency department to the ICU from January 2014 to June 2015. We defined severe sepsis as patients with SOFA score 2 points worse than sepsis onset before. Gender, age, APACHE2, SOFA, time from sepsis onset to our center visits, and time from sepsis onset to the first antibiotic administration, were examined.

**Results**: The number of patients was 60: non-survivor 18(30 %), and survivor 42(70 %). Table 2 showed characteristics of each group.

As APACHE2 or SOFA was bad, life prognosis was bad. As the definitive treatment or the first antibiotic administration was earlier, life prognosis was better.

Table 3 showed the way patients' visited the center.

**Conclusions**: The number of patients was 60: non-survivor 18(30 %), and survivor 42(70 %).

Table 2 showed characteristics of each group.

As APACHE2 or SOFA was bad, life prognosis was bad. As the definitive treatment or the first antibiotic administration was earlier, life prognosis was better.

Table 3 showed the way patients' visited the center.

**Table 2:** **(abstract P5).** Patients characteristics

	Non-survive 18 cases (%)	Survive 42 cases (%)	
Gender Male : Female	10 : 8	19 : 23	
Age	70.9 ± 11.0	73.1 ± 13.7	p = 0.543
APACHE 2	29.2 ± 8.5	17.8 ± 7.2	*p < 0.01*
SOFA score	12.4 ± 2.9	17.8 ± 7.2	*p < 0.01*
Time from sepsis onset^※^ to our center visits (days)	5.5 ± 6.3	2.9 ± 4.6	p = 0.081
Time from sepsis onset^※^ to the first antibiotic administration (days)	4.3 ± 6.0	1.9 ± 4.3	p = 0.071

※ sepsis onset: we estimated the timing of emerging organ dysfunction, such as impaired consciousness and respiratory difficulty

**Table 3:** **(abstract P5).** The way of patients’ visit our center

	Non-survive 18 cases (%)	Survive 42 cases (%)
Introduce from other hospitals after patient state worsening	9 (50)	12 (28.6)
Introduce from other hospitals immediately after patients’ visit	6 (33.3)	20 (47.6)
Directly transport to our center	3 (16.7)	8 (19.0)

## P6 Translational neuromodulation of the immune system

### Zhifeng Li, Dennis Grech, Patrick Morcillo, Alex Bekker, Luis Ulloa

#### Department of Surgery and Anesthesiology, New Jersey Medical School, Rutgers University, Newark, NJ 07103, USA

##### **Correspondence:** Luis Ulloa (luis.Ulloa@Rutgers.edu) – Department of Surgery and Anesthesiology, New Jersey Medical School, Rutgers University, NJ 07103, USA

**Background**: Sepsis, a leading cause of death in the ICU, is characterized by detrimental inflammation and multiple organ failure. We reported that electrical vagal stimulation controls inflammation and improves organ function in sepsis [1–3]. Even sepsis accounts for only 2 % of the hospitalizations; it makes up 17 % of in-hospital deaths in the US. We reasoned that nerve stimulation may control immunological stress during surgery, a major cause contributing sepsis during hospitalization.

**Materials and methods**: Here, we report a prospective double-blinded pilot trial to analyze whether intraoperative transdermal nerve stimulation prevents trauma, physiological and immunological stress during surgery.

**Results**: Transdermal nerve stimulation was performed with electroacupuncture on anesthetized patients, and blood samples were collected under anesthesia to avoid any placebo interference. Subjects with electroacupuncture required 60 % less postoperative analgesic, but they had pain scores similar to that in the control patients. Electroacupuncture prevented postoperative hyperglycemia and attenuated serum ACTH in the older and heavier group of patients. From an immunological perspective, electroacupuncture did not affect the protective immune responses to surgical trauma including the induction of IL6 and IL10. The most significant immunological effect of electroacupuncture was enhancing TGFβ1 production during surgery in the older and lighter group of patients.

**Conclusions**: These results suggest that intraoperative electroacupuncture can reduce postoperative use of analgesic and improve immune and stress responses to surgery in anesthetized patients.

**Acknowledgements**

Studies funded by the NIH-GM084125.

**References**

1. Ulloa L. Nat Rev Drug Discov 4: 673–684, 2006.

2. Peña G, et al. Journal of immunology 187: 718–725, 2011.

3. Torres-Rosas R, et al. Nat Med 20(3):291–5, 2014.

## P7 Pathway-level meta-analysis reveals transcriptional signature of septic shock

### Samanwoy Mukhopadhyay, Abhay D Pandey, Samsiddhi Bhattacharjee, Saroj K Mohapatra

#### National Institute of Biomedical Genomics, Kalyani, 741251, India

##### **Correspondence:** Saroj K Mohapatra (saroj.genomics@gmail.com) – National Institute of Biomedical Genomics, Kalyani, 741251, India

**Background**: Septic shock is a major cause of death among the critically ill. Incompletely understood biology has lent itself to be explored at the genome level. Availability of genome-wide expression data from different studies on septic shock empowers the quest for pathways of interest by integration and meta-analysis of multiple data sets.

**Materials and methods**: Electronic search was performed on medical literature (PubMed) and genome-wide gene expression databases (National Centre for Biotechnology Information Gene Expression Omnibus). Human studies of transcriptomic analysis of circulating leukocytes were selected. Systematic analysis was conducted to detect pathways differentially regulated in septic shock. Additionally coexpression network analysis was conducted.

**Fig. 13:**
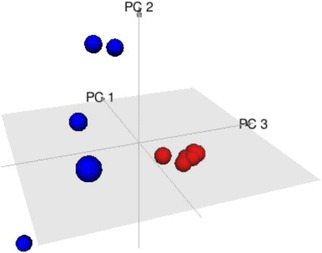
**(abstract P7).** Three dimensional plot of Principal Component Analysis (PCA) of Septic Shock (in red) and sepsis (in blue) studies. Each point represents the fold-change in gene expression of the Osteoclast Differentiation Pathway in a single study. The septic shock and sepsis studies appear well separated from each other in the three dimensional space. The sepsis studies clearly show more intra-group heterogeneity compared to septic shock studies that form a small tight cluster

**Results**: Osteoclast differentiation was consistently up-regulated in septic shock. As shown in Fig. 13, principal components analysis clearly distinguishes pathway expression in septic shock from sepsis.

**Conclusions**: A multi-gene expression signature differentiated septic shock from the milder form of sepsis. This deepens our understanding of disease biology of septic shock and is of potential value for clinical management.

**Acknowledgements**

This work was supported by an Intra-mural grant from the National Institute of Biomedical Genomics, Kalyani, India.

## P8 Antibiotic dosing in septic patients on the critical care unit - a literature review

### Julie K Wilson

#### Critical Care and Anaesthetics, Royal Marsden Hospital, London, United Kingdom

##### **Correspondence:** Julie K Wilson (juliekay85@hotmail.com) – Critical Care and Anaesthetics, Royal Marsden Hospital, London, United Kingdom

**Background**: The mortality rate for critically ill septic patients remains unacceptably high. Timely administration of appropriate antimicrobials is the cornerstone of management, and achieving therapeutic levels is essential for ensuring treatment efficacy and preventing the development of drug-resistant pathogens [1]. The purpose of this article is to review the literature pertaining to antibiotic dosing in septic patients on the intensive care unit, and to explore whether therapeutic drug monitoring (TDM) may help to optimise management of this important patient group.

**Materials and methods**: A literature search was performed using Web of Science and PubMed databases. Search terms included ‘antibiotic level*’, ‘antibiotic dos*’, ‘therapeutic drug monitoring’ and ‘sepsis’. The reference lists of selected papers were also reviewed for relevant articles.

**Results**: Critically ill septic patients exhibit a multitude of pathophysiological changes which affect drug handling, making achievement of optimum antibiotic dosing difficult [2]. Ample data is available to suggest that a worrying number of these patients do not receive therapeutic antibiotic doses [3–5]. Notably, the ‘Defining Antibiotic Levels in Intensive Care Unit Patients’ study measured serum concentrations of β-lactams in 384 patients across 68 hospitals in 10 countries. In 16 % of patients concentrations did not exceed the minimum inhibitory concentration, and this correlated with a poorer clinical outcome.[6] Similarly, a study measuring serum β-lactam concentrations in 80 patients found sub-therapeutic levels in the majority [7], and a 2014 study of 30 septic patients found linezolid levels to be sub-therapeutic in 63 % and toxic in 7 % [8]. Several studies suggest that TDM may lead to significant improvements in drug concentrations [9–10], although relatively few randomised controlled trials (RCT) have been conducted. One RCT compared TDM with standard dosing in 41 patients treated with piperacillin/tazobactam. Adequate drug concentrations were found in a significantly higher proportion of patients in the intervention than the control group. Mortality, however, did not differ significantly (although this was not a primary endpoint, and the study was not powered to detect this) [11]. Similar findings were reported from an RCT comparing TDM with standard dosing in 32 patients with neutropenic sepsis treated with piperacillin/tazobactam [12].

**Conclusions**: Based on the available evidence, it would seem that a worryingly high proportion of patients are receiving inadequate antibiotic doses, increasing the likelihood of treatment failure and the development of drug-resistance. Therapeutic drug monitoring offers a potential solution. A number of studies have demonstrated a benefit in pharmacokinetic/pharmacodynamic parameters. It remains unclear whether this translates to an improvement in clinical outcomes, and further research is required.

**References**

1. Timsit JF, Perner A, Bakker J, Bassetti M, Benoit D, Cecconi M, Curtis JR, Doig GS, Herridge M, Jaber S et al. Year in review in Intensive Care Medicine 2014: III. Severe infections, septic shock, healthcare-associated infections, highly resistant bacteria, invasive fungal infections, severe viral infections, Ebola virus disease and paediatrics. Intensive Care Med. 2015;41:575–588.

2. Roberts JA, Norris R, Paterson DL, Martin JH. Therapeutic drug monitoring of antimicrobials. Br J Clin Pharmacol. 2012;73:27–36.

3. Tsai D, Lipman J, Roberts JA. Pharmacokinetic/pharmacodynamics considerations for the optimization of antimicrobial delivery in the critically ill. Curr Opin Crit Care. 2015;21:412–420.

4. Varghese JM, Roberts JA, Lipman J. Pharmacokinetics and pharmacodynamics in critically ill patients. Curr Opin Anaesthesiol. 2010;23:472–478.

5. Felton TW, Hope WW, Roberts JA. How severe is antibiotic pharmacokinetic variability in critically ill patients and what can be done about it? Diagn Microbiol Infect Dis. 2014;79:441–447.

6. Roberts JA, Paul SK, Akova M, Bassetti M, De Waele JJ, Dimopoulos G, Kaukonen KM, Koulenti D, Martin C, Montravers P et al. DALI: defining antibiotic levels in intensive care unit patients: are current β-lactam antibiotic doses sufficient for critically ill patients? Clin Infect Dis. 2014;58:1072–1083.

7. Taccone FS, Laterre PF, Dugernier T, Spapen H, Delattre I, Wittebole X, De Backer D, Layeux B, Wallemacq P, Vincent JL et al. Insufficient β-lactam concentrations in the early phase of severe sepsis and septic shock. Crit Care. 2010;14:R126.

8. Zoller M, Maier B, Hornuss C, Neugebauer C, Döbbeler G, Nagel D, Holdt LM, Bruegel M, Weig T, Grabein B et al. Variability of linezo! lid concentrations after standard dosing in critically ill patients: a prospective observational study. Crit Care. 2014;18:R148.

9. Duszynska W, Taccone FS, Hurkacz M, Kowalska-Krochmal B, Wiela-Hojeńska A, Kübler A. Therapeutic drug monitoring of amikacin in septic patients. Crit Care. 2013;17:R165.

10. Tröger U, Drust A, Martens-Lobenhoffer J, Tanev I, Braun-Dullaeus RC, Bode-Böger SM. Decreased meropenem levels in Intensive Care Unit patients with augmented renal clearance: benefit of therapeutic drug monitoring. Int J Antimicrob Agents. 2012;40:370–372.

11. De Waele JJ, Carrette S, Carlier M, Stove V, Boelens J, Claeys G, Leroux-Roels I, Hoste E, Depuydt P, Decruyenaere J et al. Therapeutic drug monitoring-based dose optimisation of piperacillin and meropenem: a randomised controlled trial. Intensive Care Med. 2014;40:380–387.

12. Sime FB, Roberts MS, Tiong IS, Gardner JH, Lehman S, Peake SL, Hahn U, Warner MS, Roberts JA. Can therapeutic drug monitoring optimize exposure to piperacillin in febrile neutropenic patients with haematological malignancies? A randomized controlled trial. J Antimicrob Chemother. 2015;70:2369–2375.

## P9 Pandemic of *Escherichia coli* clone O25: H4-ST131 producing CTX-M-15 extended spectrum- β- lactamase- as serious cause of multidrug resistance extraintestinal pathogenic *E. coli* infections in India

### Savita Jadhav, Rabindra Nath Misra, Nageswari Gandham, Kalpana Angadi, Chanda Vywahare, Neetu Gupta, Deepali Desai

#### Department of Microbiology, Dr. D.Y.Patil Medical College, Hospital and research Centre (Dr D.Y.Patil Vidyapeeth Pune) Pimpri-Pune 411018, India

##### **Correspondence:** Savita Jadhav (patilsv78@gmail.com) – Department of Microbiology, Dr. D.Y.Patil Medical College, Hospital and research Centre ( Dr D.Y.Patil Vidyapeeth Pune) Pimpri-Pune 411018, India

**Background**: Antimicrobial resistance has emerged as an important determinant of outcome for patients in the intensive care unit (ICU). In recent years infections due to extraintestinal pathogenic *Escherichia coli* (ExPEC) predominantly *E.coli* sequence type 131 (ST131) is of great concern due to significant morbidity and mortality [2] a major clone linked to the spread of the CTX-M-15 extended spectrum- β- lactamase (ESBL) resistance is growing concern in the ICU patient’s population as it has been directly related with fluoroquinolone resistance and coresistance to aminoglycosides and trimethoprime-sulfamthoxazole, consequently delays in appropriate therapy, higher costs, and increased use of “last resort” antimicrobials e.g. carbapenemase to treat life threatening infections [3,4]. *E. coli* ST131 lineage among ExPEC is leading cause of community as well as hospital acquired urinary tract infections (UTIs) and also frequently encountered in soft tissue infections, blood stream infections (BSIs) and neonatal meningitis [1–4]. It is of major awareness to investigate its prevalence in countries such as India and to determine antibiotic resistance, virulence factors, associated clinical risk factors and potential genetic architecture of *E. coli* ST131.

**Materials and methods**: 314 phenotypically ESBL producing ExPEC were isolated from various clinical samples admitted in various ICUs, received in the clinical microbiology laboratory of tertiary care hospital India. Molecular genotyping were confirmed by allele-specific PCR targeting the rfbO25 subgroup gene locus, molecular detection of metallobetalactamases by using genes bla OXA, bla KPC, bla VIM, and bla NDM-1. Rep-PCR fingerprinting was done for phylogenetic analysis [1–5].

**Results**: Of the total of 314 phenotypically ESBL producing ExPEC, 180 (57.32 %) were positive for O25:H4-ST131 CTX-M-15 of which 155 (86.11 %) co-resistant to aminoglycosides, fluroquinilones 162 (92 %) and co-trimaxazole 138 (76.66 %) and 53 (29.44 %) were MBL positive by molecular detection. 70.34 % were from Urinary tract infections and 15.95 % were from BSIs and 10.19 % were from soft tissue infections while 3.50 % were from neonatal meningitis. Living in long-term care facility was positively associated with clinical isolates of ST131 *E. coli* while bacteremia caused by this clone was associated with secondary bacteremia from spontaneous focal infections. Nitrofurantoin found 100 % susceptible in all urinary isolates. Rep-PCR fingerprinting were distinct from UPEC isolated from global origins. pap, sfa, aer, hly were detected predominant virulence factor.

**Conclusions**: Our Meticulous analysis form significant baseline data-set towards understanding of *E. coli* clone O25: H4-ST131 producing CTX-M-15 from India. Such observations is necessary throughout the country to control this worsen situation for implementation of best infection control practices.

**References**

1. Johnson JR, Brian J, Connie C, Kuskowski MA, Mariana C: Escherichia coli sequence type ST131 as the major cause of serious multidrug- resistant E. coli infections in the United States. Clin. Infect. Dis.2010, 51:286–294.

2. Jadhav S, Hussain A, Devi S, Kumar A, Parveen S, Gandham N, Wieler LH, Ewers C, Ahmed N: Virulence characteristics and genetic affinities of multiple drug resistant uropathogenic Escherichia coli from a semi urban locality in India. PLoS One 2011, 6:e18063.

3. Clermont O, Dhanji H, Upton M, Gibreel T, Fox A, Boyd D, Mulvey MR, Nordmann P, Ruppe E, Sarthou JL, Frank T, Vimont S, Arlet G, Branger C, Woodford N, Denamur E. 2009. Rapid detection of the O25b-ST131 clone of Escherichia coli encompassing the CTX-M-15- producing strains. J. Antimicrob. Chemother. 64:274–277.

4. Nicolas-Chanoine MH, Blanco J, Leflon-Guibout V, Demarty R, Alonso MP, Canica MM, Park Y-J, Lavigne J-P, Pitout J, Johnson JR. 2008. Intercontinental emergence of Escherichia coli clone O25:H4- ST131 producing CTX-M-15. J. Antimicrob. Chemother. 61:273–281.

5. Hussain A, Ewers C, Nandanwar N, Guenther S, Jadhav S, Wieler LH, Ahmed N. 2012. Multiresistant uropathogenic Escherichia coli from a region in India where urinary tract infections are endemic: genotypic and phenotypic characteristics of sequence type 131 isolates of the CTXM- 15 extended-spectrum-_-lactamase-producing lineage. Antimicrob. Agents Chemother. 56:6358–6365.

## P10 Detection and characterization of meningitis using a DDA-based mass spectrometry approach

### Anahita Bakochi, Tirthankar Mohanty, Adam Linder, Johan Malmström

#### Department of Clinical Sciences, Division of Infection Medicine, BMC B14, Lund University, SE-221 85, Lund, Sweden

##### **Correspondence:** Tirthankar Mohanty (tirthankar.mohanty@med.lu.se) – Department of Clinical Sciences, Division of Infection Medicine, BMC B14, Lund University, SE-221 85, Lund, Sweden

**Background**: Acute bacterial meningitis (ABM) is a serious and often life threatening disease. Due to the proximity of inflammation to the brain, it can cause substantial neurological sequelae in survivors. The severity of illness and the required treatment vary according to the pathogen. In most cases it is difficult to distinguish between ABM and other types of central nervous system infections, including those caused by viruses. Therefore, early diagnosis and an effective treatment regimen are still perceived as major challenges.

**Materials and methods**: Cerebrospinal fluid (CSF) from patients suffering from ABM (n = 26), viral meningitis (VM) (n = 19), Lyme neuroborreliosis (n = 7) and headaches without any infections (n = 38) were grouped on the basis of the disease and analyzed using tandem mass spectrometry (LC-MS/MS) in data-dependent-acquisition (DDA) mode.

**Results**: A unique protein profile was found in each patient group along with a few commonly up-regulated proteins. 59 proteins were found to be affected in ABM, 22 proteins in VM and 7 in neuroborreliosis (p ≤ 0.05). A difference in the protein profile in between patient groups was also observed. Neutrophil proteins such as neutrophil elastase (NE) and myeloperoxidase (MPO) were up regulated only in case of ABM in comparison with the other groups. This is in accordance with the fact that neutrophils are the prime cells that are involved in the defense against bacteria.

**Conclusions**: Our findings suggest that by using DDA-based mass spectrometry, a unique pathogen-specific protein profile of the CSF can be detected during meningitis. This may lead to early diagnosis and better treatment options.

## P11 Diagnostic usefulness of lipid profile and procalcitonin in sepsis and trauma patients

### Dimple Anand^1^, Seema Bhargava^1^, Lalit Mohan Srivastava^1^, Sumit Ray^2^

#### ^1^Department of Biochemistry, Sir Ganga Ram Hospital, New Delhi-110060, India; ^2^Department of Critical Care and Emergency Medicine, Sir Ganga Ram Hospital, New Delhi-110060, India

##### **Correspondence:** Dimple Anand (dimplemicro85@gmail.com) – Department of Biochemistry, Sir Ganga Ram Hospital, New Delhi-110060, India

**Background**: Despite advances in medicine, identification of sepsis from non-infectious systemic inflammatory conditions such as trauma, pancreatitis remains a challenging task for clinicians. Sepsis and trauma are both found to be associated with hypocholestrolemia and inflammation. Various studies have assessed and documented the prognostic role of hypolipidemia and procalcitonin in diagnosis and prognosis of sepsis; but in the rural setting, estimation of PCT is not easily available. Hence, we elucidated the usefulness of serum lipid profile as compared to procalcitonin in diagnosis of sepsis and trauma in the ICU.

**Materials and methods**: Serum total cholesterol (TC), high-density lipoprotein cholesterol(HDL-C), low-density lipoprotein cholesterol(LDL-C) and PCT were measured in 121 community acquired sepsis patients and 31 trauma patients at admission to ICU. SPSS version 17 was used for statistical analysis. Mann Whitney U test was applied to determine the significance between two groups and receiver operating characteristic (ROC) curves were plotted; best cut off points were derived. Sensitivity, specificity, positive predictive value (PPV) and negative predictive value (NPV) were calculated.

**Results**: Median levels of TC, HDL-C, LDL-C were significantly lower whereas PCT levels were significantly higher in sepsis group as compared to trauma group (Table 4).

**Fig. 14:**
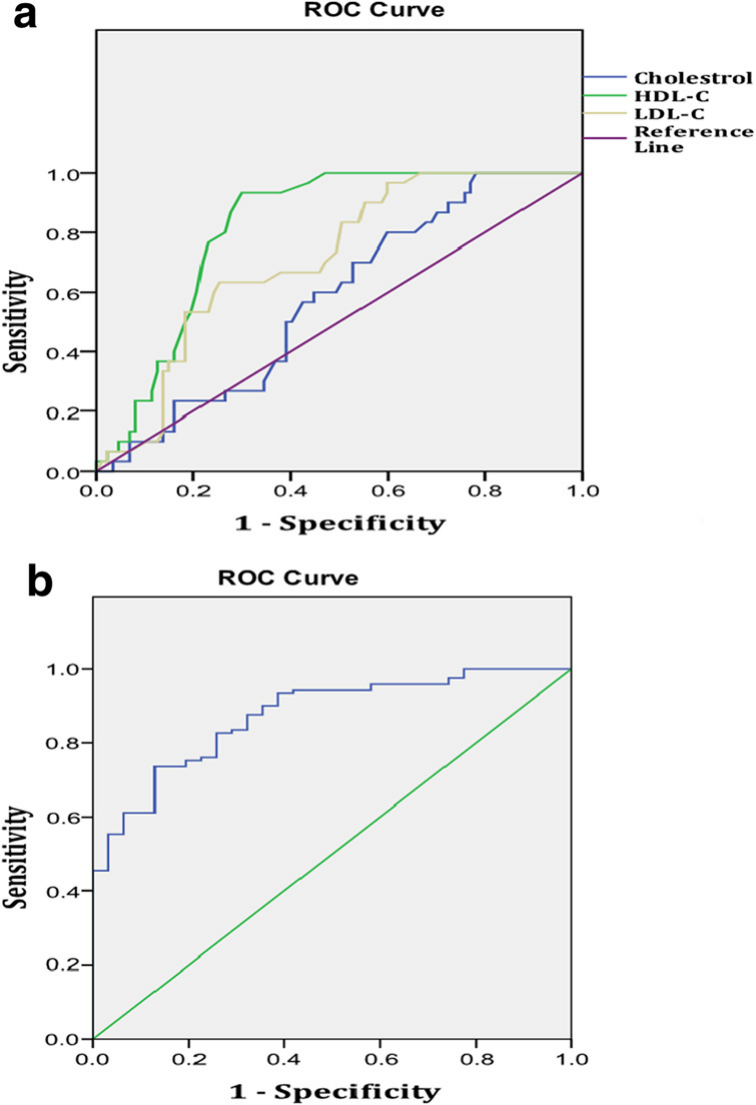
**(abstract P11).** Receiver Operating Characteristic analysis Curve for Lipid profile and Procalcitonin in sepsis versus trauma patients. **a** Lipids. **b** Procalcitonin

Among sepsis group, 33 patients had HDL below detection limit and 18 patients had LDL below detection limit. Further, ROC curve analysis between sepsis and trauma patients demonstrated a significant area under the curve (AUC) for TC (0.661; 95 % CI: 0.56-0.74), HDL-C (0.814; 95 % CI: 0.74-0.88), LDL-C (0.735; 95 % CI: 0.64-0.82), PCT (0.873; 95 % CI: 0.81-0.93). At a cutoff point of 97.5 mg/dL, TC demonstrated a sensitivity of 81 % and specificity of 50 %. However, HDL-C at cut off 22.5 mg/dL demonstrated 90 % sensitivity, 70 % specificity. LDL-C at cut off 52 mg/dL demonstrated similar sensitivity of 76 % with specificity of 63 %. PCT showed best cut off at 4.72 ng/ml with 73 % sensitivity, 87 % specificity. Correlation analysis demonstrated a moderate negative correlation of PCT with TC(r = −0.507, p = 0.004) and HDL-C(r = −0.373, p = 0.039) in trauma and LDL-C in sepsis (r = −0.283, p = 0.003) Fig. 14.

**Conclusions**: Amongst the markers evaluated, PCT and HDL-C are the most effective in differentiating sepsis from trauma. At the same time, the significance of correlations of PCT with cholesterol fractions is maximum with HDL in trauma patients and LDL in sepsis patients. Hence, in the rural setting where PCT estimation is not easily available, cholesterol fractions may be used to aid diagnosis.

**Table 4:** **(abstract P11).** Median (interquartile range) of lipid profile and procalcitonin levels in sepsis and trauma group at the time of admission

Parameter	Sepsis Group (n = 121)	Trauma Group(n = 31)	p value
Cholesterol (mg/dl)	96 (66–141)	124 (99–162)	0.006
HDL (mg/dl)	16 (8–25)	29 (25–37)	0.001
LDL (mg/dl)	31 (19–51)	58.5 (34–77.2)	<0.001
PCT (ng/ml)	16.6 (4–92)	1.25 (0.44-3.97)	<0.001

p value <0.005 considered significant

## P12 Heparin – a novel therapeutic in sepsis?

### Jane Fisher^1^, Peter Bentzer^2^, Adam Linder^1^

#### ^1^Department of Clinical Sciences, Division of Infection Medicine, BMC B14, Lund University, SE-221 85, Lund, Sweden; ^2^Department of Anesthesia and Intensive Care, Skåne University Hospital, SE-221 85, Lund, Sweden

##### **Correspondence:** Adam Linder (adam.linder@med.lu.se) – Department of Clinical Sciences, Division of Infection Medicine, BMC B14, Lund University, SE-221 85, Lund, Sweden

**Background**: Heparin binding protein (HBP) is released from neutrophils early in the immune response, which leads to sepsis. It is a very promising predictive biomarker for the development of sepsis and plasma levels are associated with development of organ failure in patients. Heparin is a drug typically used as an anticoagulant however it has recently been shown to affect many different systems, including inflammation. HBP binds very strongly to heparin and some studies have suggested that the use of heparin can have beneficial effects in sepsis. Therefore we hypothesized that HBP plays a causative role in the development of vascular leakage and AKI in sepsis and that heparin derivatives can be used to block these effects.

**Materials and methods**: *In-vitro*, cultured endothelial and renal epithelial cells were treated with recombinant HBP. Trans-endothelial electrical resistance (TEER) was used to evaluate permeability of endothelial cell monolayers. IL-6 ELISA was used to evaluate HBP-induced inflammation in renal cells. Recombinant HBP was also injected into mice and scanning electron microscopy used to evaluate plasma leakage and inflammation. Administration of different heparin derivatives was used to block HBP-induced effects on permeability and IL-6 release in vitro and plasma leakage in the lungs and kidneys *in-vivo*.

**Results**: TEER showed that HBP significantly (P < 0.001) increased the permeability of endothelial cell monolayers. HBP also induced the release of IL-6 from renal cells (P < 0.01). This suggests that HBP plays causative roles in the development of vascular leakage and renal inflammation. HBP was also injected into mice and scanning electron microscopy revealed that it induced plasma leakage in the lungs and the kidneys. Administration of different heparin derivatives blocked the ability of HBP to induce permeability (p < 0.001) and IL-6 release *in-vitro* (P < 0.001) and plasma leakage in the lungs and kidneys *in-vivo*.

**Conclusions**: Heparin binding protein (HBP) appears to play a causative role in sepsis and heparin compounds may be potential new therapeutics in its treatment.

## P13 Hypothalamic impairment is associated with vasopressin deficiency during sepsis

### Luis Henrique Angenendt da Costa^1^, Nilton Nascimentos dos Santos Júnior^1^, Carlos Henrique Rocha Catalão^1^, Maria José Alves da Rocha^2^

#### ^1^Department of Neurosciences and Behavioral Sciences, Ribeirão Preto Medical School, University of São Paulo, Ribeirão Preto, Brazil; ^2^Department of Morphology, Physiology and Basic Pathology, School of Dentistry of Ribeirão Preto, University of São Paulo, Ribeirão Preto, Brazil

##### **Correspondence:** Luis Henrique Angenendt da Costa (luis.angenendt@gmail.com) – Department of Neurosciences and Behavioral Sciences, Ribeirão Preto Medical School, University of São Paulo, Ribeirão Preto, Brazil

**Background**: Clinical and experimental studies have shown many hormonal alterations during sepsis. Considering vasopressin (AVP) secretion, in the early phase of sepsis its plasma concentration is increased. However, during the pathophysiological process the plasma levels remain inadequately low, despite of persistent hypotension. One of the hypotheses suggested for this relative deficiency is the apoptosis of vasopressinergic neurons in hypothalamus. Our objective was to identify elements involved in the hypothalamic cellular death and evaluate the modifications of glial cells and blood–brain-barrier (BBB) during sepsis.

**Materials and methods**: Male Wistar rats (250-300 g) were submitted to sepsis by cecal ligation and puncture (CLP) or non-manipulated (naïve), as control and then divided in two groups. In the first one, they were perfused and brains were collected for immunohistochemistry. In another one they were decapitated for blood collection and further plasma interferon-gama (IFN-γ) analysis by ELISA. Brain was also removed for apoptosis-related proteins expression analysis in the hypothalamus or in the supraoptic (SON) and paraventricular (PVN) nuclei by western blot. A third one was separated for the investigation of BBB permeability.

**Results**: Despite of increased immunostaining for CD8 and MHC-I in the SON of septic animals, we did not find evidence of cell death mediated by immune cells. In the SON and PVN of septic animals, the expression of proteins involved in the activation of the extrinsic apoptosis pathway (tBID, cleaved caspase-8) was not altered, whereas anti-apoptotic factors related to the intrinsic pathway (BCL-2, BCL-xL) were decreased. In the SON of these animals, microglia assumed a morphology related to its activation, associated with the increase of plasma IFN-γ. There was a transitory breakdown of BBB in hypothalamus after 6 hours following CLP.

**Conclusions**: The results indicate that the intrinsic apoptosis pathway seems to be responsible for the cell death observed in vasopressinergic nuclei and this condition is associated with microglial activation and BBB leaking.

## P14 Presepsin (soluble CD14 subtype) is a dependable prognostic marker in critical septic patients

### Alfredo Focà^1^, Cinzia Peronace^1^, Giovanni Matera^1^, Aida Giancotti^1^, Giorgio Settimo Barreca^1^, Angela Quirino^1^, Maria Teresa Loria^1^, Pio Settembre^1^, Maria Carla Liberto^1^, Bruno Amantea^2^

#### ^1^Institute of Microbiology, Department of Health Sciences, University “Magna Graecia” of Catanzaro, Catanzaro, Italy; ^2^Intensive Care Unit, Department of Health Sciences, “Magna Graecia” University of Catanzaro, Catanzaro, Italy

##### **Correspondence:** Giovanni Matera (gm4106@gmail.com) – Institute of Microbiology, Department of Health Sciences, University “Magna Graecia” of Catanzaro, Catanzaro, Italy

**Background**: Since decades worldwide investigators are searching reliable sepsis markers for prognostic and diagnostic evaluation of critically ill patients. Our group has already assessed sCD25 and IL-10 as dependable sepsis markers. Recently presepsin (soluble CD14 subtype, sCD14-ST) has been shown to increase in plasma of patients with sepsis. Here we evaluated the role of presepsin (PRES) in predicting outcome of critical septic patients; SOFA, procalcitonin (PCT) and C-reactive protein (CRP) were also assessed in parallel.

**Materials and methods**: Critical patients admitted to the Unit of Intensive Care (ICU) of the University Hospital of Catanzaro (Italy) were sequentially enrolled into this observational prospective study, if a sepsis was clinically suspected; healthy volunteers were also included as controls. The SOFA score was assessed at the time of ICU admission. Clinical and microbiological data including blood culture were collected periodically. Based on 28 days survival, subjects were stratified in survivors and non-survivors. Plasma and serum samples were collected at multiple time points; samples were tested for presepsin (PATHFAST Presepsin assay), procalcitonin (VIDAS BRAHMS PCT), C-reactive protein and IgG4 (BNTM II System immunonephelometry). A statistical analysis was carried out by ANOVA plus PLSD Fisher’s test.

**Fig. 15:**
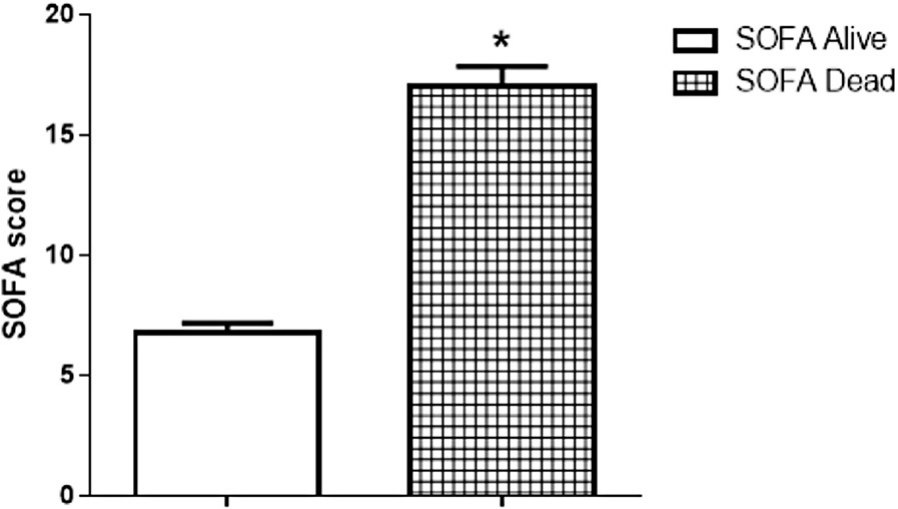
**(abstract P14).**

**Results**: At the admission SOFA scores were found significantly higher (p < 0.001) in nonsurvivor patients vs. survivor subjects (Fig. 15).

**Fig. 16:**
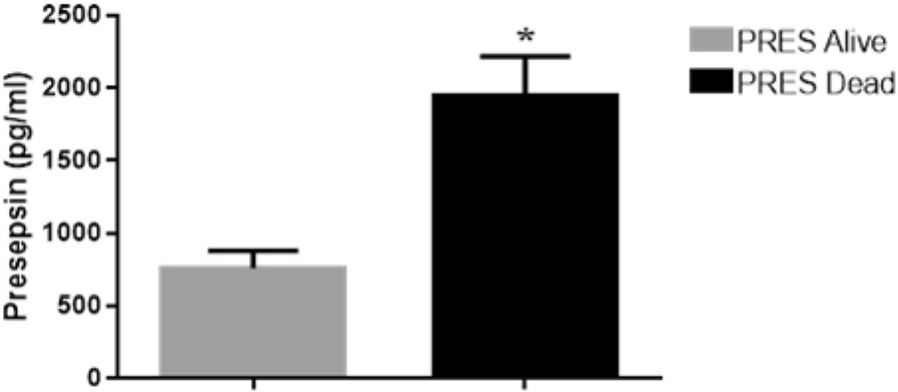
**(abstract P14).**

Levels of presepsin were significantly more elevated at T-0 (p = 0.0007) (Fig. 16), at T-1 (p < 0.0001) and at T-2 (p < 0.0001) in non-survivors vs. survivors at the same time. Presepsin concentrations were found significantly increased at T-0 (p = 0.0073), T1 (p = 0.0111) and T2 (p = 0.0167) in blood culture-positive in comparison to culture-negative patients at the same time.

In culture-positive patients at T-0 both PCT and CRP levels, were found significantly enhanced vs. culture-negative with p values of 0.0353 and 0.0331, respectively. Presepsin data from dead/alive individuals were subjected to ROC analysis, which demonstrated an excellent accuracy and significant AUROCC (p < 0.0001) at all the times evaluated; CRP did not exhibit a significant AUROCC at any time and PCT showed a significant AUROCC (p < 0.0001) only at T-2. Levels of IgG4 at T-0 were significantly (p < 0.04) higher in non-survivors when compared with survivors and with healthy subjects.

**Conclusions**: Presepsin may increase SOFA contribution to management decisions and level of treatment in studied patients. Markers belonging to acquired immunity deserve further evaluation. Presepsin appeared a reliable prognostic tool and revealed also an interesting diagnostic value.

## P15 Safety and efficacy of gelatin-containing solutions versus crystalloids and albumin - a systematic review with quantitative and qualitative summaries

### Christiane Hartog^1,2^, Claudia Moeller,^1^ Carolin Fleischmann,^1,2^ Daniel Thomas-Rueddel,^1^ Vlasislav Vlasakov,^1^ Bram Rochwerg,^3^ Philip Theurer,^1^ Konrad Reinhart,^1,2^

#### ^1^Department for Anesthesiology and Intensive Care, Jena University Hospital, Jena, Germany; ^2^Center for Sepsis Control and Care, Jena University Hospital, Jena, Germany; ^3^Department of Medicine (Division of Critical Care), McMaster University, Hamilton, Ontario, Canada

##### **Correspondence:** Christiane Hartog (Christiane.Hartog@med.uni-jena.de) - Department for Anesthesiology and Intensive Care, Jena University Hospital, Jena, Germany

**Background**: Gelatin solutions are regarded as fluid alternatives to crystalloids or albumin to treat hypovolemia in the ICU. However, little is known about their safety and efficacy. In parts of the world their use increased as hydroxyethyl starches were restricted due to safety concerns.

**Materials and methods**: Systematic review and meta-analysis of randomised and non-randomised controlled trials. Data sources included the Cochrane Central Register of Controlled Trials, Medline, Embase, Indmed, MedCarib, AJO, AIM, IMEMR, WHOLIS, LILACS, WPRIM, IMSEAR, Google Scholar, Grey Literature, German National Library, the German Pharmacovigilance Database and Clinical Trials Registries, until August 2015. Trials were included if they compared gelatin with either crystalloid or human albumin for the treatment of hypovolemia and reported data on defined outcomes. Uncontrolled studies which reported “extravascular uptake” were also included. No language was excluded. Risk of bias was assessed using the Cochrane tool for RCTs and the Ottawa Newcastle scale for observational studies. Certainty of evidence was assessed using the GRADE methodology.

**Results**: 60 studies were included, consisting of 30 RCTs with 3629 patients, 8 non-randomised studies with 10827 patients and 22 animal studies. For those receiving gelatin, the relative risks (RR) were as follows: for mortality (RR 1.18, 95 % CI 0.98-1.41, 16 RCTs, 2525 patients; low certainty in evidence), requiring allogenic blood transfusion (RR 1.25, 95 % CI 0.95-1.64, 8 RCTs, 744 patients; low certainty in evidence), acute kidney injury (RR 1.32, 95 % CI [0.61, 2.87], 3 RCTs, 212 patients, very low certainty in evidence), anaphylactic adverse effects (RR 2.18, 95 % CI 0.87-5.44, 3 RCTs 872 patients, very low certainty of evidence). Mean crystalloid-to-colloid ratio was 1.44 (±0.31, 6 RCTs). Four observational controlled studies (9403 patients, low risk of bias) all found an increased risk for acute kidney injury (AKI) or need for renal replacement therapy (RRT). Reported elimination deficits indicating extravascular uptake ranged from 17 to 31 % (3 non-randomised, uncontrolled cohort studies, 95 subjects). Eleven of thirteen animal studies provided evidence of histopathological changes in the kidneys after gelatin administration.

**Conclusions**: Despite the poor quality of published evidence, the meta-analysis of RCTs demonstrated a trend towards increased mortality and bleeding while observational studies suggested an increased rate of renal failure. Plasma expansion with gelatin is not associated with a relevant benefit. In the absence of evidence from adequately designed RCTs showing these solutions to be safe and effective, gelatin plasma expanders should no longer be used outside the research setting.

**Funding**

Carolin Fleischmann was partly funded by the German Ministry of Education and Research (grant number 01 E0 1002).

## P16 Immunomodulatory properties of peripheral blood mesenchymal stem cells following endotoxin stimulation in an equine model

### Anna E. Smith, Sandra D. Taylor

#### Department of Veterinary Clinical Sciences, College of Veterinary Medicine, Purdue University, West Lafayette, Indiana, USA

##### **Correspondence:** Sandra D. Taylor (taylo248@purdue.edu) – Department of Veterinary Clinical Sciences, College of Veterinary Medicine, Purdue University, West Lafayette, Indiana, USA

**Background**: Bacterial sepsis in humans is a common cause of illness and death worldwide, accounting for 60-80 % of deaths in developing countries and 20-30 % of deaths in the United States [1–3]. Overwhelming inflammation associated with Gram-negative sepsis (endotoxemia; lipopolysaccharide [LPS]) can lead to organ failure and death, and there is a critical need to identify therapy that will target this inflammation. Recently, horses have been identified as an important, emerging model for human sepsis, given that sepsis occurs naturally and is a leading cause of illness and death in both neonatal and adult horses [4]. Similar to sepsis in humans, horses develop sepsis-associated acute lung injury, septic peritonitis from bowel disruption, and bacteremia [4–8]. Studies have shown that mesenchymal stem cells (MSC) can decrease lymphocyte proliferation and downregulate pro-inflammatory cascades [9,10]. Specifically, MSC have been shown to secrete IL-6 and PGE2 in response to TNF-α, which results in decreased lymphocyte proliferation and thus, downregulation of pro-inflammatory cascades [11,12]. Importantly, equine MSC are easily harvested from peripheral blood, which allows for simple isolation and minimal donor morbidity [13]. We hypothesized that addition of equine peripheral blood MSC (PB-MSC) to cultures of equine mononuclear cells would result in increased production of IL-6, PGE2 and TXA2, and inhibition of TNF-α and IL-8 production following LPS stimulation.

**Materials and methods**: We investigated the immunomodulatory properties of PB-MSC following stimulation with 100 pg/mL of lipopolysaccharide (LPS). Equine PB-MSC were added to equine monocyte cultures at ratios of 1:1, 1:2, 1:5, 1:10, and 1:100 (monocytes:PB-MSC), and supernatants were collected 12 hours after the onset of LPS treatment. Culture supernatant concentrations of the pro-inflammatory cytokines TNF-α, IL-6 and IL-8 and the eicosanoids PGE2 and TXA2 were evaluated by enzyme-linked immunosorbent assays (ELISA).

**Fig. 17:**
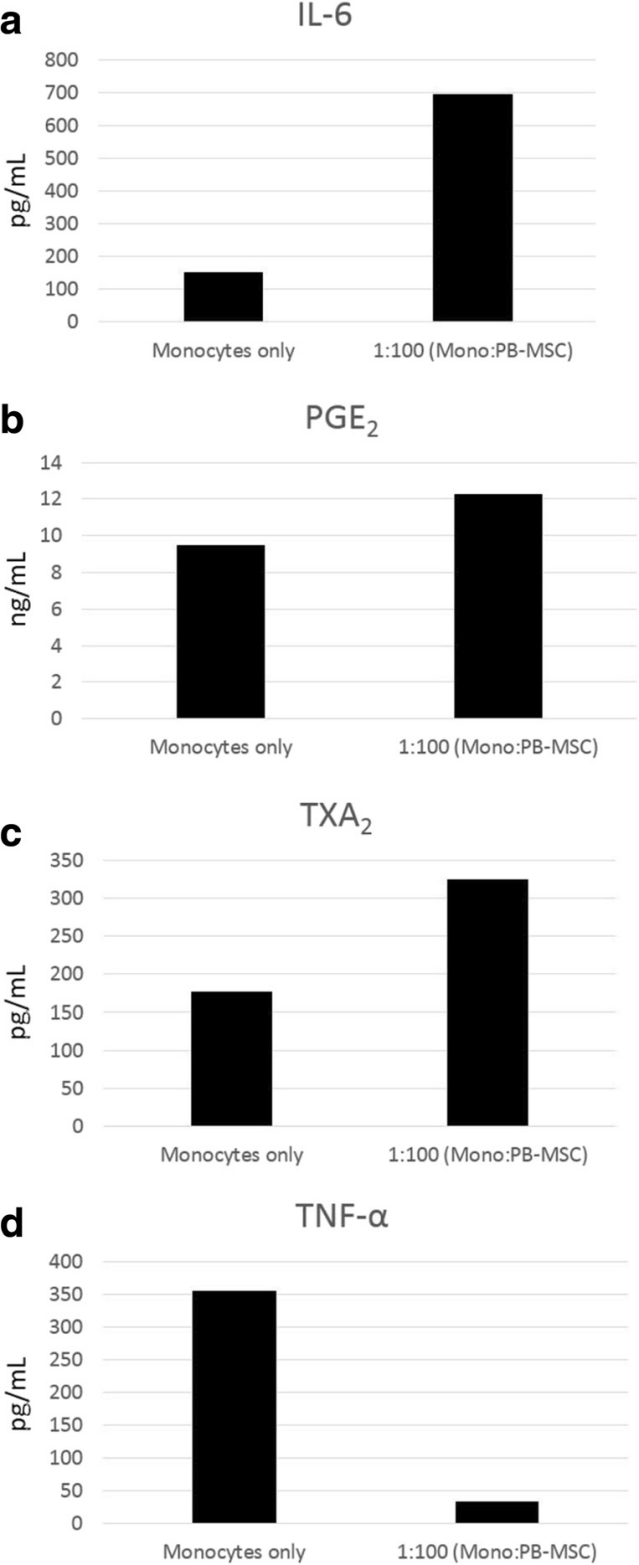
**(abstract P16).**

**Results**: As expected, the addition of equine PB-MSC to equine monocyte cultures resulted in increased concentrations of IL-6, PGE2 and TXA2, and decreased concentrations of TNF-α at 12 hours post-LPS stimulation (Fig. 17). There was no effect of equine PB-MSCs on IL-8 concentrations 12 hours post-LPS stimulation (data not shown).

**Conclusions**: Equine PB-MSC appear to respond in vitro to LPS-stimulated equine monocytes by facilitating increases in cytokines that suppress lymphocyte proliferation and inhibit selective pro-inflammatory cytokines. Subsequent studies will evaluate lymphocyte responses *in-vitro*, as well as the safety and efficacy of equine PB-MSC in an *in-vivo* equine model of endotoxemia. These results have important implications for treatment of sepsis in horses and other species.

**Acknowledgements**

The authors sincerely thank Anisa Dunham and Natalia Hernandez Diaz for technical assistance. Funding was provided by the Purdue University College of Agriculture AgSEED grant and the Ralph W. and Grace M. Showalter Research Trust.

**References**

1. Cheng AC, West TE, Limmathurotsakul D, Peacock SJ: Strategies to reduce mortality from bacterial sepsis in adults in developing countries. PLoS Med 2008, 5(8): e175.

2. Mathers C: World Health Organization methods and data sources for country-level causes of death 2000 – 2012. Global Health Estimates Technical Paper 2014.

3. Dombrovskiy VY, Martin AA, Sunderram J, Paz HL: Rapid increase in hospitalization and mortality rates for severe sepsis in the United States: a trend analysis from 1993 to 2003. Crit Care Med 2007, 35: 1244–1250.

4. Roy MF: Sepsis in adults and foals. Vet Clin North Am Equine Pract 2004, 20: 41–61.

5. Koterba AM, Brewer BD, Tarplee FA: Clinical and clinicopathological characteristics of the septicaemic neonatal foal: review of 38 cases. Equine Vet J 1984, 16(4): 376–382.

6. Dunkel B, Dolente B, Boston RC: Acute lung injury/acute respiratory distress syndrome in 15 foals. Equine Vet J 2005, 37(5): 435–440.

7. Van Hoogmoed L, Rodger LD, Spier SJ, Gardner IA, Yarbrough TB, Snyder JR: Evaluation of peritoneal fluid pH, glucose concentration, and lactate dehydrogenase activity for detection of septic peritonitis in horses. J Am Vet Med Assoc 1999, 214(7): 1032–1036.

8. Theelen MJ, Wilson WD, Edman JM, Magdesian KG, Kass PH: Temporal trends in prevalence of bacteria isolated from foals with sepsis: 1979–2010. Equine Vet J 2014, 46(2): 169–173.

9. Shin S, Kim Y, Jeong S, Kim I, Lee W, Choi S: The therapeutic effect of human adult stem cells derived from adipose tissue in endotoxemic rat model. Int J Med Sci 2013, 10(1): 8–18.

10. Wannemuehler TJ, Manukyan MC, Brewster BD, Rouch J, Wang Y, Meldrum DR: Advances in mesenchymal stem cell research in sepsis. J Surg Res 2012, 173(1): 113–26.

11. Peroni JF, Borjesson DL:! Anti-inflammatory and immunomodulatory activities of stem cells. Vet Clin Equine 2011, 27: 351–362.

12. Aggarwal S, Pittenger MF: Human mesenchymal stem cells modulate allogeneic immune cell responses. Blood 2005, 105(4): 1815–1822.

13. De Schauwer CD, Goossens K, Piepers S, Hoogewijs MK, Govaere JL, Smits K, Meyer E, Soom AV, Van de Walle GR: Characterization and profiling of immunomodulatory genes of equine mesenchymal stromal cells from non-invasive sources. Stem Cell Res Ther 2014, 5(6): 1–13.

## P17 Frequency and outcome of early sepsis-associated coagulopathy

### Christopher Da Costa^1^, Amanda Radford^1^, Terry Lee^2^, Joel Singer^2^, John Boyd^3,4^, David Fineberg^1^, Mark Williams^1^, James A Russell^3,4^

#### ^1^Asahi Kasei Pharma America, Waltham, MA, USA; ^2^Centre for Health Evaluation and Outcome Science (CHEOS), St. Paul’s Hospital, University of British Columbia, 1081 Burrard Street, Vancouver, BC, Canada V6Z 1Y6; ^3^Centre for Heart Lung Innovation, St. Paul’s Hospital, University of British Columbia, 1081 Burrard Street, Vancouver, BC, Canada V6Z 1Y6; ^4^Division of Critical Care Medicine St. Paul’s Hospital, University of British Columbia, 1081 Burrard Street, Vancouver, BC, Canada V6Z 1Y6

##### **Correspondence:** Christopher Da Costa (cdacosta@akpamerica.com) – Asahi Kasei Pharma America, Waltham, MA, USA

**Background**: The frequency of early (within the first three days of onset) sepsis-associated coagulopathy (SAC) and its association with clinical outcomes varies depending on the definition(s) of coagulopathy. Furthermore, the frequency of SAC may have decreased with more effective use of sepsis bundles (early antibiotics, fluids, and vasopressors). Accordingly, we sought to determine the frequency and outcome of early SAC.

**Materials and methods**: We reviewed all patients admitted to the medical-surgical Intensive Care Unit (ICU) of St. Paul’s Hospital, a tertiary care hospital in Vancouver, Canada from January 2011 to July 2013. We included patients who met SAC inclusion criteria: sepsis and platelet count less than 150,000 (or a decrease of at least 30 %) and International Normalized ratio (INR) greater than 1.2 within the first three days of onset of sepsis. We assessed the presence and severity of SAC and the association of SAC with hospital mortality and need for vasopressors, ventilation and renal replacement therapy (RRT).

**Results**: Of 1,397 ICU admissions 517 had sepsis and of these, 373 (27 % of ICU admissions, 72 % of septic patients) had at least 1 INR and platelet count at any point from day 1 to 3 of ICU admission. 20 to 35 % of septic patients met various criteria for SAC. Presence of SAC at 12 hours or at 24 hours was associated with significantly increased mortality (Odds Ratio (OR) = 2.3 & 1.94 at 12 and 24 hrs respectively) and need for vasopressors (OR = 4.6 & 6.4), ventilation (OR = 1.68 & 2.13) and RRT (OR = 1.71 & 2.94) in both unadjusted and adjusted analyses. Increasing severity of the combination of abnormal INR and platelets was associated with significantly higher mortality and greater need for vasopressors and RRT but not ventilation. Increasingly abnormal INR was associated with increasing mortality in a monotonic, increasing fashion whereas only severe thrombocytopenia (platelets (<80,000) was associated with significantly increased mortality.

**Conclusions**: SAC is common (20 – 35 % of septic patients) and is associated with increased mortality and need for vasopressors, ventilation and renal replacement therapy. Accordingly, there is a need for therapies that decrease the severity of SAC to attempt to decrease mortality and organ dysfunction in sepsis.

